# Seroprevalence of transfusion-transmissible infections and evaluation of the pre-donation screening performance at the Provincial Hospital of Tete, Mozambique

**DOI:** 10.1186/1471-2334-11-141

**Published:** 2011-05-23

**Authors:** Jocelijn Stokx, Philippe Gillet, Anja De Weggheleire, Esther C Casas, Rosa Maendaenda, Adelino J Beulane, Ilhes V Jani, Solon Kidane, Carla D Mosse, Jan Jacobs, Emmanuel Bottieau

**Affiliations:** 1 Department of Clinical Sciences, Institute of Tropical Medicine (ITM), Antwerp, Belgium; 2 Provincial Hospital of Tete (PHT), Tete, Mozambique; 3 Instituto Nacional da Saude (INS), Maputo, Mozambique; 4 Direcçao Provincial da Saude (DPS), Tete, Mozambique

## Abstract

**Background:**

The World Health Organization recommends universal and quality-controlled screening of blood donations for the major transfusion-transmissible infections (TTIs): human immunodeficiency virus (HIV), hepatitis B virus (HBV), hepatitis C virus (HCV) and syphilis. The study objectives were to determine the seroprevalence of these TTIs among blood donors at the Provincial Hospital of Tete, Mozambique, and to assess the local pre-donation screening performance.

**Methods:**

All consenting voluntary and replacement candidate blood donors were consecutively included from February to May 2009. Sera of all candidates, independent of deferral by questionnaire, were submitted to screening with quality-assured rapid or simple assays for HIV, HBV surface antigen (HBsAg), HCV and syphilis. Assays locally used by the blood bank for HBV and syphilis screening were run in parallel to quality-assured external assays supplied during the study, and all discordant samples were submitted to confirmation testing in reference laboratories in Mozambique and Belgium.

**Results:**

Of 750 consenting candidates (50.5% of voluntary donors), 71 (9.5%) were deferred by the questionnaire, including 38 specifically because of risk behavior for TTI. Of the 679 non-deferred candidates, 127 (18.7%) had serological confirmation of at least one TTI, with a lower prevalence in voluntary than in replacement donors (15.2% versus 22.4%, p = 0.016). Seroprevalence of HIV, HBsAg and syphilis infections was 8.5%, 10.6 % and 1.2%. No confirmed HCV infection was found. Seroprevalence of TTIs was similar in the 38 candidates deferred for TTI risk as in the non-deferred group, except for HBsAg (26.3 % versus 10.6 %; p = 0.005). The local assays used for HBV and syphilis had sensitivities of 98.4% and 100% and specificities of 80.4% and 98.8% respectively. This resulted in the rejection of 110 of the 679 blood donations (16.2%) because of false positive results.

**Conclusions:**

The seroprevalence of TTIs after questionnaire screening is high in Tete, Mozambique, but HCV infection does not appear as a major issue. The questionnaire did not exclude effectively HIV-infected donor candidates, while the locally used assays led to unnecessary rejection of many safe donations. A contextualized questionnaire and consistent use of quality-assured assays would considerably improve the current screening procedure for blood donation.

## Background

Every year more than 90 million units of blood are collected worldwide [1]. Each transfusion carries a risk of transmitting blood-borne pathogens, including mainly human immunodeficiency virus (HIV), hepatitis B virus (HBV), hepatitis C virus (HCV) and syphilis. To improve blood transfusion safety, the World Health Organization (WHO) recommends an integrated strategy including establishment of well-organized blood transfusion services, prioritization of blood donation from voluntary non-remunerated donors, screening of donated blood for at least the four major transfusion-transmissible infections (TTI) with quality-assured assays, rational use of blood and implementation of effective quality control systems [2]. Selection of blood donors with low TTI risk followed by effective laboratory screening is the critical part of the process, since it has reduced the risk of transmission to very low levels in the past 20 years [3,4]. Nevertheless, particularly in low-resource countries, a significant proportion of donated blood remains unsafe as it is either not screened for all major TTIs or not in a quality-controlled manner [1,5].

Africa faces the highest transfusion needs in the world, but also the highest prevalence of blood-borne pathogens and the weakest transfusion programs [6]. Most blood banks in Africa are small, hospital-based and relying on an important proportion of replacement donors, in contrast with western transfusion units organized with large pools of voluntary donors [7]. In addition, recommended reference screening tests like enzyme immunoassays (EIA) or nucleic acid testing (NAT) are technically, logistically and financially still far beyond reach of many resource-constrained blood banks [8]. In such settings with limited capacity and low throughput, WHO accepts the use of rapid and simple serological assays for TTI screening, provided that they are quality-assured, locally validated and quality-controlled. Rapid test-based screening protocols tend to be used increasingly in African blood banks [9]. It seems indeed effective at least for HIV and HCV screening [10], although safety of such a strategy has been recently challenged in an international quality control survey [11]. For some experts, rapid testing has also the advantage of immediate counseling of infected candidates and referral to appropriate care, although this is not the primary objective of transfusion medicine [12].

In the province of Tete (3 million inhabitants), central region of Mozambique, prevalence of HIV and syphilis infections among pregnant women was about 13% and 7% respectively in the national sentinel surveillance round of 2007 [13]. Provincial prevalence of HBV and HCV infections was not known. In the reference provincial hospital of Tete (PHT), more than 2,500 blood transfusions were administered every year; about half of them were obtained from voluntary donors. Candidate blood donors underwent a first screening by the national questionnaire, and non-deferred candidates were then routinely screened for HIV, HBV and syphilis by means of rapid or simple assays. HIV testing was performed with internationally quality-assured assays (see definition under Methods), while HBV and syphilis infections were usually screened by a variety of brands, depending on "ad-hoc" supply of the Ministry of Health (MoH), and not always complying with international quality criteria. There was no screening for HCV at the time this study was conducted (2009).

The main objectives of this study were to determine the seroprevalence of HIV, HBV, HCV and syphilis infections in blood donors of the provincial hospital of Tete and to assess the screening performances of the national questionnaire and of the rapid assays used locally for HBV and syphilis detection.

## Methods

### Study design and population

A cross-sectional study was set up in the blood bank of the provincial hospital of Tete from February till May 2009. The study was executed by the regular blood bank staff, which got an additional training prior to the start of the study and respected the national blood transfusion guidelines. All consecutive candidate blood donors, either voluntary or replacement, were invited to participate and after signing the informed consent, enrolled. The study complied with the STROBE recommendations for reporting on cross-sectional diagnostic studies and with the STARD guidelines for comparisons between diagnostic tests [14,15].

### Questionnaire and blood sampling

All consenting candidate donors underwent first the national selection questionnaire (Table 1). In accordance with Mozambican guidelines, candidate donors were deferred when they answered "yes" to at least one question (except for the question number 7, formulated negatively, for which a "no" answer caused deferral). A donor was deferred definitively when he was older than 65 years of age (question 1), had a chronic disease (question 2) or was at risk of having a sexual transmitted infection (STI) (questions 3 to 6). He was deferred temporarily when not being knowledgeable about HIV transmission modes (question 7) or having been refused for donation in the past (question 8). Other reasons for temporary exclusion were the presence of a pathologic condition at the moment of donation (question 9-18), the presence of another condition not compatible with donation (question 19-22: pregnancy, menstruation, medication, recent vaccination or surgery), or the presence of a physical or laboratory contra-indication (items 23-28). Filling accurately this questionnaire requires at least 30 minutes, including the translation from Portuguese to the local language.

**Table 1:** Mozambican screening questionnaire for blood donation and main reasons for deferral in this study (n = 71)

Questionnaire		Number of persons excluded per question (n = 71)
**Questions for definitive exclusion**		
01	Do you have more than 65 years of age?	0 (-)
02	Do you have a disease without cure (chronic)?	2 (2.6%)
03	Were you once suspected of being infected with HIV?	1 (1.3%)
04	Did you have once hepatitis/jaundice?	4 (5.1%)
05	Did you already have once a venereal disease?	22 (28.2%)
06	Do you belong to a risk group for HIV infection (injection of drugs, sexual contacts without condom, traditional vaccines with blades used for different persons, several blood transfusions)?	6 (8.5%)

**Questions for temporary exclusion**		
07	A person with HIV can not donate blood?	4 (5.1%)
08	Were you refused before to donate blood?	1 (1.4%)
09	Do you suffer from diseases of the cardiovascular system (e. g pericardial pain)?	2 (2.6%)
10	Do you suffer from diseases of the respiratory system (e.g., asthma)?	6 (8.5%)
11	Do you suffer from diseases of the gastrointestinal system (e.g.; peptic ulcer)	1 (1.4%)
12	Do you suffer from diseases of the central nervous system (e.g. epilepsy)?	1 (1.4%)
13	Do you suffer from diseases of the muscular/skeletal system?	0 (-)
14	Do you suffer from diseases of the genital/urinary system?	0 (-)
15	Do you suffer from diseases of the endocrine system? (e.g. diabetes).	0 (-)
16	Do you suffer from diseases of the immune system? (e.g. allergy)	1 (1.4%)
17	Do you have a blood disease? (e.g. hemophilia)?	0 (-)
18	Do you have a fever?	3 (5.1%)
19	Are you pregnant /menstruating? (women)	2 (2.6%)
20	Are you taking any medication?	0 (2.6%)
21	Were you vaccinated in the last 4 weeks?	1 (1.4%)
22	Were you operated in the last 6 months?	0 (-)

**Physical examination**		
23	Control if donor has infectious diseases	0 (-)
24	Do you have a blood pressure higher than 160/110 mmHg or lower than 120/80 mmHg?	1 (1.4%)
25	Do you have a pulse higher than 120 beats per minute (bpm) or lower than 70 bpm?	0 (-)
26	Do you have a weight below 50 kg?	5 (6.4%)
27	Do you have a hemoglobin level below 12.5 g/dl?	3 (3.8%)
28	Others	5 (7.0%)

Whole blood of candidates not deferred by questionnaire was collected and serum (blood sample of 10 ml in a dry tube) was tested for HIV, HBV, syphilis and HCV (see below). After testing, all serum samples were stored at -20°C in Tete until shipment for external quality control at the "Instituto Nacional da Saude" (INS) in Maputo, Mozambique and the Institute of Tropical Medicine (ITM) in Antwerp, Belgium.

To assess the selective performance of the national selection questionnaire, whole blood of deferred candidates was also collected after informed consent and tested in the same way as for non-deferred donors.

### Rapid test screening and confirmatory procedures

For this study, and by analogy to the quality assurance process for drugs [16], we considered as "quality-assured" diagnostic tests which have been validated by the WHO (and included in the WHO bulk procurement list) or by a stringent regulatory authority, or which have received the WHO prequalification attestation.

#### HIV testing

Presence of HIV-1 and HIV-2 antibodies was screened with the quality-assured rapid test Determine HIV-1/2 (Abbott Laboratories, Illinois, USA), which has a reported sensitivity of 100% and specificity of 99.4% [17]. For study purposes and in accordance with national algorithms for diagnostic HIV testing, blood found positive was thereafter tested for confirmation with another quality-assured rapid test Uni-Gold HIV-1/2 (Trinity Biotech, Dublin, Ireland) which is 100% sensitive and specific on whole blood. Both rapid tests are in the WHO bulk procurement 2010 list for HIV test kits. Reported HIV seroprevalence corresponds to the proportion of cases tested positive with both tests [18].

#### HBV testing

Hepatitis B surface antigen (HBsAg) testing was performed with the rapid test Healthease HBsAg (Neomedic Ltd., Sea Cow Lake, South Africa) supplied by the MoH and in use in the blood bank. However, since this test lacked independent quality assurance data, another rapid test, Determine HBsAg (Abbott Laboratories, Illinois, USA), from the WHO bulk procurement 2010 list, was supplied by ITM and run in parallel during the study (reported sensitivity: 100%; specificity: 99.4% [19]). In case of discordant results, both tests were repeated to exclude manipulation errors. All sera with persisting discordance were sent to the INS in Maputo and analyzed by the EIA Murex HBsAg Version 3.0 (Abbott Diagnostics Division, Murex Biotech Limited, Dartford, UK; sensitivity: 99.7% and specificity: 99.5%). Reported HBsAg seroprevalence corresponds to the result obtained with the rapid test Determine HBsAg rapid test corrected by EIA (this procedure being further considered as our reference diagnostic method). Sensitivity and specificity of the rapid test locally used (Healthease HBsAg) were calculated with standard formulas against this reference method.

#### Syphilis testing

For the same reason as for HBV, syphilis was screened with 2 rapid plasma reagin (RPR) card tests in parallel: one used routinely in the blood bank, the RPR test AraGen (AraGen Biotech, Amman, Jordan), and the quality-assured RPR test BD Macro-Vue RPR (Becton, Dickinson and Company, Maryland, USA) supplied by the ITM for this study. In case of discordant results, both tests were repeated. Any positive RPR test was submitted in the Tete blood bank to confirmation with the quality-assured *Treponema pallidum *particle agglutination (TP°PA) test: SERODIA®- TP°PA (Fujirebio, Tokyo, Japan). Reported syphilis seroprevalence corresponds to the proportion of cases positive with the RPR test BD Macro-Vue RPR further confirmed with SERODIA®- TP°PA (considered as our reference diagnostic method). Sensitivity and specificity of the rapid test locally used (Aragen) were calculated with standard formulas against this reference method.

#### HCV testing

HCV testing, not performed routinely in the provincial hospital, was added to the pre-donation serological screening during the study period. HCV antibodies were detected trough the quality-assured rapid test, SD BIOLINE HCV (Standard Diagnostics, Kyonggi-do, Korea). Reactive samples were re-analyzed at the INS with the EIA Murex anti-HCV version 4.0 (Abbott Diagnostics Division, Murex Biotech Limited, Kyalami, Republic of South Africa) as well as with the Line ImmunoAssay (LIA) confirmatory test INNO-LIA HCV (Innogenetics, Ghent, Belgium). Molecular confirmation and genotyping was planned in ITM for samples found positive with LIA. Reported HCV seroprevalence corresponds to the proportion of cases confirmed positive with LIA.

### Management of blood donations and candidate donors

Blood reactive to any of the locally used or ITM-supplied screening tests was discarded. All donors found positive with any screening test were offered specific counseling about the need for further confirmatory workup and were informed when the confirmatory results were available (immediately for syphilis, several months later for HBV and HCV infections after workup in Maputo and Antwerp). Individuals with confirmed serological diagnosis of TTI were evaluated and managed according the current standard of care in Mozambique. Donors found with positive Determine HIV-1/2 but negative Uni-Gold HIV-1/2 (discordant results) were immediately re-tested and in case of persistent discordance were referred to the HIV counseling program where both tests were repeated within one month according to the national algorithm. Donors with confirmed HIV infection were immediately referred to the HIV care and treatment program.

### Quality control of the laboratory results

Internal quality controls were performed weekly. External quality control took place in the INS, where all sera with discordant rapid test results for HBsAg were analyzed with EIA (as already mentioned). The INS retested also 10% of the samples for which both HBsAg rapid tests had given concordant results. All samples reactive to HCV rapid testing as well as 10% of the non-reactive samples were re-checked at the INS. External quality control of screening and confirmatory results was also performed for all four pathogens under investigation at the ITM in Belgium.

### Sample size and statistical analysis

Since the HCV seroprevalence had never been investigated in Tete and was estimated to be rather low (about 2% according to the few available regional data [20,21]), the sample size calculation targeted specifically this condition. We calculated that 750 participants had to be included to obtain with a confidence interval (CI) of 95% a level of accuracy of +/- 1% around the expected HCV prevalence of 2%.

Measures of prevalence were expressed in percentages and reported with 95% confidence intervals. Comparisons between subgroups were done using the Pearson Chi-Square test. All tests were 2-tailed and P < 0.05 indicated statistical significance.

### Ethical issues

All participants were explained the purpose of the study and signed an informed consent. The study followed the national blood donation procedures except that three additional quality-assured rapid tests and three confirmatory tests were performed on collected blood. There was no additional risk for the blood donors. Participants were offered information on the confirmatory procedures and referred to standard care in case infection was confirmed. In Belgium, the study was approved by the Institutional Review Board of the ITM and by the Ethical Review Committee of Antwerp University. In Mozambique approval was given by the National Bioethical Committee and the Ministry of Health.

## Results

### Seroprevalence of transfusion-transmissible infections

Of 758 candidate donors, 750 consented to participate in the study (88.9% males, mean age 27 ± 9 years), including 379 (50.5%) voluntary donors. Seventy-one (9.5%) candidates were deferred by means of the screening questionnaire. The rate of rejection was similar among voluntary (31/379; 8.2%) and replacement donors (40/371; 10.8%).

The demographic data and laboratory results of the 679 non-deferred candidates are detailed per type of donors in Table 2. At least one transfusion-transmitted infection was serologically confirmed in 127 (18.7%) of them. Prevalence of at least one TTI was significantly lower in voluntary than in replacement donors (15.2% versus 22.4%; p = 0.016). Seroprevalence of HIV, HBV, HCV and syphilis infection was 8.5% (95% CI: 6.6-10.9%), 10.6% (95% CI: 8.4-13.2%), 0.0% and 1.2% (95% CI 0.5-2.3%) respectively (Table 2). There were eight donors found with HIV-HBV co-infection and two co-infected with HIV and syphilis. Prevalence of each infection was lower in voluntary than in replacement donors, but the differences were not statistically significant (Table 2).

**Table 2:** Frequency of positive serological tests for, and confirmation of, HIV, HBsAg, HCV and syphilis infections in non-deferred (voluntary and replacement) blood donor candidates at the provincial hospital of Tete, Mozambique

	Total donors (n = 679)	Voluntary donors (n = 348)	Replacement donors (n = 331)	*P*
**Demographic data**				
Male	609 (89.7%)	310 (89.1%)	299 (90.3%)	0.59
Age 25-65 years	331 (48.7%)	124 (35.6%)	207 (62.5%)	< 0.001

**HIV testing**				
Positive Determine HIV-1/2 rapid test	73 (10.8%)	31(8.9%)	42 (12.7%)	0.14
Positive Determine HIV-1/2 and Uni-Gold HIV-1/2 rapid tests	58 (8.5%)	24 (6.9%)	34 (10.3%)	0.12

**HBV testing (HBsAg)**				
Positive Determine HBsAg rapid test	69 (10.2%)	30 (8.6%)	39 (11.8%)	0.2
Positive Healthease HBsAg rapid test	190 (28.0%)	97 (27.9%)	93 (28.1%)	1.0
Positive Determine HBsAg rapid test corrected by Murex HBsAg EIA (INS)	72 (10.6%)	30 (8.6%)	42 (12.7%)	0.13

**HCV testing**				
Positive SD Bioline HCV rapid test	6 (0.9%)	4 (1.1%)	2 (0.6%)	0.69
Positive SD Bioline HCV rapid test confirmed by INNO-LIA (INS)	0	0	0	-

**Syphilis testing**				
Positive BD Macro-Vue RPR card test	12 (1.8%)	4 (1.1%)	8 (2.4%)	0.25
Positive AraGen RPR card test	16 (2.4%)	5 (1.4%)	11 (3.3 %)	0.13
Positive BD Macro-Vue RPR card tests confirmed by SERODIA TP°PA	8 (1.2%)	2 (0.6 %)	6 (1.8%)	0.17

**At least one confirmed transfusion-transmissible infection**	127 (18.7%)	54 (15.2%)	74 (22.4%)	0.016

Note: EIA denotes Enzyme ImmunoAssay; LIA Line ImmunoAssay; INS "Instituto Nacional da Saúde" (Maputo); RPR: rapid plasma reagin; TP°PA *Treponema pallidum *particle agglutination.

For HCV infection, six samples (1.03%) were reactive to the HCV rapid test in Tete, but none of them was found positive with the EIA testing or with the confirmation LIA testing performed in Maputo (4 negative and 2 intermediate results). Therefore, no sample had to be submitted to molecular confirmation in Antwerp. Of note, during the quality control of the negative HCV rapid test results, 5 samples (on 69 tested) were reactive to EIA, but none of them was thereafter confirmed by LIA.

Quality control with screening and confirmatory tests in Maputo and in Antwerp did not show any discordant result with those obtained in Tete, except for HbsAg results (see below).

### Performance of screening questionnaire

The main reasons for deferral of the 71 candidate donors are shown in Table 1. Thirty-eight candidate donors (54%) were excluded because of STI antecedents (answered "yes" to question 5; n = 22) or presumed risk for STI (answered "yes" to questions 3, 4, 6 or 8, or answered "no" to question 7; n = 16). Four candidates were excluded because of a history of jaundice (question 4), with 3 of them actually found positive for HBsAg. Candidates deferred because of STI risk were more often older than 25 years (25/38, 65.8% versus 331/679, 48.7%; *P *= 0.04).

When comparing the seroprevalence of TTIs between donors deferred because of sexual risk behavior (n = 38) and non-deferred donors (n = 679), no difference could be found, except for HBsAg positivity, more frequently observed in the former group (26.3% vs. 10.6%, p = 0.003).

### Comparisons between screening tests for HBsAg and syphilis

A large discordance was observed between the results of the two rapid tests for HBsAg screening: 122 samples non-reactive to the Determine HBsAg rapid test reacted to the Healthease HBsAg rapid test. Three of them were found positive with the EIA performed in Maputo, and all three samples were in fact reactive to the Determine HBsAg rapid test when it was re-performed in Maputo (same lot number as in Tete), suggesting that performing or reporting errors had occurred in Tete. All 119 other samples reactive to Healthease HBsAg (but not reactive to Determine HBsAg) were negative when tested with EIA in Maputo (Table 3). On the other hand, one sample reactive to the Determine HBsAg rapid test but not reactive to the Healthease HBsAg rapid test was finally confirmed as a true positive by EIA. Therefore, when using the results of the Determine HBsAg rapid test corrected by EIA as reference (Table 3), we found that the sensitivity of the Healthease HBsAg rapid test was 98.6% (95%CI: 92.8-99.8%) and the specificity 80.4% (95%CI: 79.7-80.5%).

**Table 3:** Comparison of Healthease HBsAg rapid test results with reference testing (Determine HBsAg rapid test corrected by Murex HBsAg)

Test results	Reference testing: Determine HBsAg corrected by Murex HBsAg		Total
		
	Positive	Negative	
Healthease HBsAg positive (n)	71	119	190
Healthease HBsAg negative (n)	1	488	489

Total	72	607	679

For syphilis, there were less discordant results: four samples reactive to the AraGen RPR test did not react to the BD Macro-Vue RPR test. None of them was confirmed by SERODIA-TP°PA (Table 4). No sample negative with Aragen RPR tested positive with BD Macro-Vue RPR. Of the 12 samples reactive to the BD Macro-vue RPR, 8 were confirmed by SERODIA-TP°PA. When compared to the reference method, the AraGen RPR test had a sensitivity of 100% and a specificity of 98.8% (95% CI: 98.0-99.6)

**Table 4:** Comparison of AraGen RPR card test with reference testing (BD Macro-Vue RPR card test corrected by SERODIA®-TP°PA)

Test results	Reference testing: BD Macro-Vue RPR corrected by SERODIA®-TP°PA		Total
		
	Positive	Negative	
RPR AraGen positive (n)	8	8	16
RPR AraGen negative (n)	0	663	663

Total	8	671	679

Finally, 253 of the 679 (37.3%) donor candidates not initially deferred were excluded from donation because their blood was reactive to at least one screening test. However, 110 (16.2%) had been in fact needlessly rejected because of false positive results due to the use of non-quality-assured screening tests in the blood bank.

## Discussion

In this study, we found a high prevalence of transfusion-transmissible infections in blood donor candidates of the provincial hospital of Tete, Mozambique, and in particular in replacement donors. Seroprevalence of HIV, HBsAg, and syphilis was 8.5%, 10.6 %, and 1.2% respectively, and no donor was found with confirmed HCV infection. We observed also that the questionnaire had a selective value for candidate donors with HBV infection, but did not discriminate those infected with HIV or syphilis. Finally, the rapid test for HBV screening used by the national program had an unacceptably low specificity, resulting in a high proportion of donations needlessly rejected.

Several limitations are to be mentioned. The study was conducted according to the routine practice of the blood bank, with no additional staff or equipment. Full time supervision (24 hours a day, 7 days a week) by the main investigator was not possible, and errors in performing or reporting may have occurred. Also, rapid tests were compared in parallel and in a non-blinded way, with some risk of "cross-influence" on the results. As mentioned, sophisticated testing could only be performed in Maputo, representing considerable logistical efforts. For this reason, reference testing was not performed on the whole sample set but purposely restricted to the samples with discordant results in Tete, to all samples reactive to HCV rapid test and to the subset of positive and negative samples sent for quality control. Some erroneous results of rapid testing might therefore have been missed, although the very satisfactory results of the quality control suggest that our findings were robust. Finally, the adequacy of clinical indication for transfusion has not been investigated here; also the study was not designed to assess accurately the further uptake of candidate donors diagnosed with any TTI within clinical care programs.

Little is known on the seroprevalence of transfusion-transmissible infections among blood donors in Mozambique. In one such study conducted in Maputo in 2004, the HIV prevalence was higher (13.8%) [20] than here, reflecting probably the regional differences in "background" HIV endemicity in Mozambique. In fact, our findings are in line with those of the most recent population survey in the province of Tete (7.0% in adults in 2009) [22]. Regarding the prevalence of HBsAg carriage in our study population, it was similar to that observed in blood donors in Maputo (9.3%) [20] and in neighboring Malawi (8.1%) [23]. For syphilis, little is known among Mozambican blood donors. The prevalence we observed is much lower than that obtained during the last sentinel surveillance round of 2007 among pregnant women in Tete [13], but this may be partly explained by the differences in diagnostic design: RPR testing is indeed less specific in pregnant women [24], and no confirmation test was used. Finally, no HCV infection was confirmed in this study, suggesting that HCV is not a major health problem in the province. This is in line with results from Maputo and Malawi where the prevalence of active HCV infection confirmed by molecular methods was below 1% in both sites [20,23]. Of note, anti-HCV EIA disclosed also about 10% of false positive results that could not be confirmed with the INNO- LIA HCV, like observed elsewhere in Africa [20,23,25]. Since this study, HCV screening of blood donation has been implemented in Tete, and since 2010 in the rest of the country as well, in accordance with the 2008 WHO recommendation for universal HCV testing [1]. However cost-effectiveness remains an issue for low-resource settings with very low HCV prevalence [25,26]. In the blood bank of Tete, only the cost of the rapid HCV screening tests represents an additional amount of at least 7,000 USD annually, for a few prevented infections. Although innovative testing strategies could be explored in Tete [27], it is likely that HCV screening could become really cost-effective only if the prices of available rapid tests were drastically reduced.

The proportion of young voluntary donors was rather high in comparison with other African countries where it reaches only 20-30% of the blood donors [28,29]. Not surprisingly, they were less often infected by blood-borne pathogens [28-30]. Although already important, the efforts provided by the Tete blood bank managers to select low-risk groups are for sure still worth being amplified.

In contrast, the selection power of the questionnaire was somehow disappointing. Hepatitis B carriage was rather well discriminated (mainly because of the question 4), but HIV infection and syphilis were as prevalent in the deferred as in the non-deferred group. We did not investigate specifically this issue, but reasons contributing to this low performance may include the rather time-consuming, vague and redundant questionnaire design, favoring a speedy and inadequate question-answer process, the lack of privacy to answer honestly to some delicate questions or the difficulties for both the candidate donors and the blood bank staff in interpreting/translating in local language some questions not immediately obvious or not culturally adapted to rural Africa (questions 3, 5-8, 14). We suggest to both the local and national health authorities to design and implement a shorter, simpler and more contextualized screening questionnaire, and to re-evaluate its discriminative value in similar real-life settings.

This study has also highlighted that pre-donation screening of TTI with rapid tests may be safe in settings where there is no alternative, if all quality procedures are respected. In transfusion medicine, false negative screening results are of course the most harmful, and this was fortunately not observed here with the tests locally used. However, blood is a life-saving product extremely difficult to obtain. The harm of deferring unnecessarily a large number of potential donors and of communicating to them incorrect test results should not be underestimated. In collective donations where blood is often tested subsequently, destroying large amounts of blood bags is a huge waste of time, energy and money in already overstretched settings. Erroneous laboratory results may go undetected in the absence of quality control, and may be due to multiple causes such as intrinsic weaknesses of a test not produced under strict quality regulations, inadequate conservation and manipulation, or inappropriate local validation. Quality assurance and control are therefore critical issues; surprisingly however, it has been difficult during our research to find independent and transparent technical information on laboratory test performances, on appropriate conditions for purchase, transport and conservation, or on the processes of test validation, prequalification, approval or recommendation. There is an urgent need to make this information more accessible for appropriate decision making particularly in resource-constrained blood banks.

## Conclusion

This study demonstrates the substantial risk of transfusion-transmissible infections in Tete Province, Mozambique, except for hepatitis C. Targeting of donors with lower risk profile, contextualization of the screening questionnaire, constant supply of quality-assured rapid or simple screening tests and rigorous quality control are critical elements at reach of resource-constrained settings to improve access to safe blood.

## Competing interests

The authors declare that they have no competing interests.

## Authors' contributions

PG, SK and EB were involved in the study design; JS, PG, ADW, ECC, RM, AJB and CDM were involved in the laboratory and clinical supervision in Tete; JS, PG, IR and JJ were involved in the laboratory work in Maputo and Antwerp; JS, PG, ADW, JJ and EB were involved in analysis and interpretation of the data. Finally all authors read and approved the final manuscript.

## Pre-publication history

The pre-publication history for this paper can be accessed here:

http://www.biomedcentral.com/1471-2334/11/141/prepub
